# Romantic attachment, infertility-related stress, and positive body image of women dealing with infertility

**DOI:** 10.3389/fpsyg.2022.1067970

**Published:** 2023-01-06

**Authors:** Vincenzo Calvo, Chiara Fusco, Camilla Pellicelli, Chiara Masaro

**Affiliations:** ^1^Department of Philosophy, Sociology, Pedagogy and Applied Psychology, University of Padua, Padua, Italy; ^2^Department of Psychology, University of Milano-Bicocca, Milan, Italy

**Keywords:** romantic attachment, infertility-related stress, positive body image, infertility, medically assisted reproduction (MAR)

## Abstract

**Introduction:**

Infertility is a condition that can affect the physical, emotional, social, and relational well-being of women. Women’s bodies seem to assume a crucial relevance as part of the experience of infertility and its treatments. An extended body of literature supports the role of romantic attachment orientations in facing infertility-related stress. However, the association between romantic attachment orientations, infertility-related stress, and women’s body image has not been explored.

**Methods:**

This cross-sectional study aimed to investigate the role of romantic attachment and infertility-related stress concerning positive body image in 113 women dealing with infertility. Data were analyzed with correlation and mediation path analyses.

**Results:**

Results showed that high levels of attachment anxiety, attachment avoidance, and infertility-related stress were negatively associated with positive body image. Path analyses indicated that positive body image may be directly associated with romantic attachment anxiety. The negative association of attachment avoidance with body image appeared to be mediated by infertility-related stress.

**Discussion:**

Findings suggest that romantic attachment insecurities and infertility-related stress are significantly associated with a worsened body image in infertile women. Implications for future research are discussed.

## 1. Introduction

Infertility is a condition that globally affects 8–12% of reproductive-aged couples (Vander Borght and Wyns, 2018). It is defined as “a disease characterized by the failure to establish a clinical pregnancy after 12 months of regular, unprotected sexual intercourse or due to an impairment of a person’s capacity to reproduce either as an individual or with his/her partner” (Zegers-Hochschild et al., 2017). Infertility may represent a potentially stressful condition, especially for women (El Kissi et al., 2013; Pasch and Sullivan, 2017; Zurlo et al., 2019).

About half of infertile women seek medically assisted reproduction (MAR) treatments to achieve a clinical state of pregnancy (Boivin et al., 2007). Such application involves a significant amount of economic, physical, and psychological resources (Cousineau and Domar, 2007) and may contribute to the stressful impact of infertility, along with the invasiveness of MAR techniques and the uncertainty related to the chance of success of these treatments (Pasch and Sullivan, 2017).

Several studies have investigated the psychological implications of infertility as a stressful medical condition that can have a role in the psychological adjustment of women experiencing infertility. The effects of infertility and stress concern dyadic adjustment, couple satisfaction (Van Der Merwe and Greeff, 2015; Vizheh et al., 2015; Omani-Samani et al., 2018), and sexual domain (Wischmann, 2010; Tao et al., 2011; Peterson, 2015). Concerning psychosocial well-being, increased levels of anxiety and depression among infertile women have been observed (Newton et al., 1999; Zurlo et al., 2017, 2019; Chaves et al., 2019). Besides, it has been found that infertility seems to be related to negative perceptions of women’s body image (Younesi and Salajegheh, 2001; Karamidehkordi and Roudsari, 2014; Ozen et al., 2019), which is defined as the set of attitudes, behaviors, self-perceptions, and feelings about one’s own body and physical appearance (Cash and Pruzinsky, 2002; Cash et al., 2004).

In the context of body image studies, the main research focus has been on the negative components of this construct (van den Brink et al., 2016), such as body dissatisfaction or body shape preoccupations. However, the positive psychology movement has emphasized the importance of exploring positive body image as well (Avalos et al., 2005), which does not entail the absence of negative body image but the presence of positive feelings and favorable opinions about the body (Tylka, 2011). For instance, body appreciation is considered a relevant component of positive body image, as it refers to the set of attitudes, thoughts, and behaviors that define the acceptance, love, and respect for one’s own body and the rejection of media aesthetic standards as the only possible model of beauty (Avalos et al., 2005; Tylka and Wood-Barcalow, 2015). Positive body image may be a particularly salient construct for women facing infertility since it may be related to other relational domains such as sexual functioning and intimacy (van den Brink et al., 2016).

Indeed, the experience that women report regarding their bodies has emerged to be significantly related to their psychological adjustment (Akhondi et al., 2011; Karamidehkordi and Roudsari, 2014). In the context of infertility, the body appears to be simultaneously both what prevents women from conceiving and the target of MAR techniques (Cousineau and Domar, 2007; Cipolletta and Faccio, 2013). MAR techniques include procedures that are physically and psychologically highly intrusive (Cousineau and Domar, 2007) and can have implications in terms of grief experiences (McBain and Reeves, 2019), such as loss of bodily integrity and loss of control over the body, as well as identity-related issues (Bell, 2019). Women are more frequently exposed to highly invasive treatments during MAR programs, regardless of the diagnostic status (Halcomb, 2018; Bell, 2019). Accordingly, women tend to report negative psychological outcomes, such as sexual dysfunction (Wischmann, 2010), low self-esteem, depression, and anxiety (Wichman et al., 2011; El Kissi et al., 2013; Nik Hazlina et al., 2022).

Moreover, infertility has emerged to have a negative role in women’s body image (Younesi and Salajegheh, 2001). The distortion of body image appears to be a common phenomenon observed among women, who tend to report negative perceptions of their bodies (Akhondi et al., 2011). This detrimental effect, in turn, seems to be negatively associated with dyadic adjustment, sexual functioning (Karamidehkordi and Roudsari, 2014), and the psychological well-being of women facing infertility (Hwang, 2017). A recent study (Ozen et al., 2019) confirmed that the body image of infertile women is significantly negatively affected by infertility status when compared to highly fertile women (with more than 5 children). This seems to occur, in particular, in sociocultural contexts where having many children is highly valued and encouraged (Ozen et al., 2019).

Based on the above considerations, the present study aimed to explore the potential associations between infertility-related stress and the body image of women dealing with infertility.

### 1.1. The role of romantic attachment, infertility related-stress, and positive body image of women dealing with infertility

The attachment theory (Bowlby, 1969; Hazan and Shaver, 1987) represents a framework that allows an integration of the multiple aspects involved in the infertility experience. In the context of romantic relationships, literature has widely acknowledged the conceptualization of love as an attachment bond and romantic partners as reciprocal attachment figures (Hazan and Shaver, 1987). Within romantic attachment theory, infertility may represent a threat to individual attachment security that may activate women’s attachment-oriented behaviors and emotion regulation strategies (Mikulincer and Shaver, 2016; Moura-Ramos et al., 2017; Calvo et al., 2021). Extant studies within the framework of attachment theory have shown that attachment orientations may have a significant role in the psychological adjustment of women during adulthood (Calvo et al., 2014, 2022; Calvo and Bianco, 2015) and, specifically, in their level of infertility-related stress (Mikulincer and Shaver, 2005; Lowyck et al., 2009; Simpson and Rholes, 2010).

Infertility-related stress has been defined as a multifactorial construct referring to the multi-facets domains related to infertility and subjectively perceived as sources of stress (Newton et al., 1999). These domains include the social, romantic, and sexual dimensions, the need for parenthood, and the rejection of a child-free lifestyle (Newton et al., 1999; Zurlo et al., 2017). In this respect, attachment theory can connect the romantic relationship, conceptualized as an attachment bond (Hazan and Shaver, 1987, 1994; Fraley and Shaver, 2000), to the perception of infertility-related stress, which can be conceptualized as a psychological outcome of infertility in terms of attachment-related threat (Bayley et al., 2009; Van den Broeck et al., 2010; Donarelli et al., 2012).

A significant positive association has been found between high levels of attachment avoidance and attachment anxiety – dimensions that define attachment insecurity (Brennan et al., 1998) – and greater levels of infertility-related stress (Bayley et al., 2009; Donarelli et al., 2012). Mikulincer et al. (1998) investigated the contribution of adult attachment style to the adjustment to infertility, finding significant differences among attachment categories: women with secure attachment reported more general well-being and less distress than women with anxiety/avoidant attachment orientations. Besides, associations between women’s attachment dimensions and their psychological adjustment were observed in the literature (Amir et al., 1999). Other studies highlighted correlations between attachment anxiety and infertility-related stress experienced by women (Bayley et al., 2009) and higher levels of psychophysical health reported by individuals securely attached (Lowyck et al., 2009). Interestingly, a recent study revealed that anxious romantic attachment is associated with lower levels of infertility-related quality of life and that avoidant attachment is negatively related to the success of assisted reproductive technology treatment for women (Renzi et al., 2020).

Besides, romantic attachment theory provides a conceptual framework to understand how the infertility condition may affect women’s body image. Body image is a socially constructed phenomenon, reciprocally related to the way adults experience their interactions with others (Cash and Pruzinsky, 2002; Cash et al., 2004; Satinsky et al., 2012), and also significantly related to how individuals experience their romantic relationships (McKinley, 1999; Cash et al., 2004). Over the years, many studies have demonstrated the salience of adult attachment orientations in shaping individual perceptions of romantic relationships (Feeney, 2016). These two viewpoints combined have led to the proposal that secure attachment may promote more favorable body image development, whereas insecure orientations may foster less positive and dysfunctional body image attitudes (Cash et al., 2004; Cash, 2011; Tantleff-Dunn and Lindner, 2011).

Yet, a relatively small number of studies have empirically verified the association between women’s romantic attachment and their body image. The extant studies have highlighted a negative association between romantic attachment anxiety with several core facets of body image attitudes, whereas the role of attachment avoidance is less clear (Cash et al., 2004; McKinley and Randa, 2005; Koskina and Giovazolias, 2010; Mili and Raakhee, 2015; van den Brink et al., 2016). Importantly, Cash et al. (2004) found that anxious romantic attachment was the strongest predictor of body image dysfunctionality, among other attachment variables, for women. Romantic attachment anxiety was also related to women’s greater body image dissatisfaction, distress, appearance investment, and body satisfaction (Cash et al., 2004; McKinley and Randa, 2005). Moreover, attachment anxiety was also recently found to be negatively related to women’s body appreciation (van den Brink et al., 2016). Furthermore, a recent study investigated the relationship between adult attachment and body-image avoidance, highlighting that body-image avoidance was associated with women’s anxious attachment (Salcuni et al., 2021).

### 1.2. The current study

The study aimed to investigate the interplay between romantic attachment, infertility-related stress, and positive body image in women dealing with infertility. According to the literature, adult attachment is described as an individual characteristic relatively stable over time in adulthood (Zhang and Labouvie-Vief, 2004), not being substantially affected by the condition of infertility (Fistikci et al., 2014), and significantly influencing the well-being, adjustment, and functioning of infertile persons (Mikulincer et al., 1998). Therefore, we hypothesized that higher levels of attachment insecurity orientations (attachment avoidance and, principally, attachment anxiety) could be associated with higher infertility-related stress and lower levels of positive body image in women facing infertility.

Consistently with the theoretical premises, which link together these three constructs, and adult attachment theory, we then developed an integrative mediating model encompassing romantic attachment, infertility-related stress, and positive body image. The model hypothesized that infertility-related stress could mediate the association between romantic attachment and positive body image of women experiencing infertility. We expected insecure attachment orientations to show both a negative direct and indirect association with women’s positive body image, given the probable role of romantic attachment orientations in the experience of infertility-related stress.

## 2. Materials and methods

### 2.1. Participants

One hundred thirteen women dealing with infertility participated in the study. Participants’ ages ranged from 20 to 54 years, with a mean age of 36.7 years (*SD* = 6.0). The mean level of their education was 15.2 years (*SD* = 3.7). The average duration of infertility was 4.2 years (*SD* = 4.0). Infertility was female-related in 36 participants (31.9%), male-related in 27 (23.9%), combined in 25 (22.1%), and unexplained in 25 (22.1%). All participants were involved in a heterosexual relationship at the time of completion of the survey. The mean length of the relationship was 9.9 years (*SD* = 5.8), the mean duration of current cohabitation was 6.9 years (*SD* = 4.5) and most of the participants were married (64.6%). Most of the women reported being childless (*N* = 87, 77%), while 26 (23%) had already one or more children at the moment of the data collection.

### 2.2. Procedure

Women who had experienced a diagnosis of infertility (i.e., they, their respective partners, or the couple were diagnosed with infertility) were recruited *via* social media (Facebook) advertisements requesting to participate in a study about attachment, infertility, and body image. The announcements were posted in various discussion groups and internet blogs about parenthood, infertility, and medically assisted reproduction (MAR). Ads were also distributed using a chain sampling method to people involved in infertility experiences. Women willing to participate in the study received an internet link to the online anonymous survey designed to collect socio-demographic and infertility-related information, as well as responses to validated questionnaires for the assessment of romantic attachment orientations, infertility-related stress, and positive body image.

The study was approved by the Ethical Committee of Psychological Research at the University of Padova (protocol id. 3,438).

### 2.3. Measures

#### 2.3.1. Romantic attachment

The attachment orientation of participants was assessed using the Italian version (Calvo, 2008) of the Experiences in Close Relationships - Revised questionnaire (ECR-R; Fraley et al., 2000), which evaluates two central underlying dimensions of attachment insecurity: Attachment anxiety and Attachment avoidance. Attachment anxiety refers to the individual’s insecurity and fear of being abandoned in close relationships, vulnerability to rejection and loss, and tendency to use hyperactivating strategies in attachment-related experiences (Mikulincer and Shaver, 2016). Attachment avoidance indicates the individual’s propensity of being in discomfort with intimacy, closeness, and dependence, and a tendency to employ deactivating defensive strategies in dealing with attachment issues. The ECR-R comprises 36 items scored on a Likert scale from 1 to 7 (example item, “I prefer not to show a partner how I feel deep down”). Higher total scores indicate greater levels of anxiety and/or avoidance, and therefore lower degrees of attachment security. In the present study, Cronbach’s alpha was 0.87 for attachment anxiety, and 0.91 for attachment avoidance scores.

#### 2.3.2. Infertility-related stress

The Fertility Problem Inventory–Short Form (FPI-SF; Zurlo et al., 2017) was used to measure significant domains of infertility-related stress. The FPI-SF is shortened version and adaptation of the Fertility Problem Inventory (Newton et al., 1999); it comprises 27 items on a 6-point Likert scale, ranging from 1 (Strongly disagree) to 6 (Strongly agree), evaluating four factors of infertility-related stress: Social concern (10 items), Need for parenthood (6 items), Rejection of childfree lifestyle (6 items), and Couple’s relationship concern (5 items). A composite measure of global infertility-related stress is derived by summing the scores of all five scales. All the internal consistency coefficients (Cronbach’s alpha) in the study were highly satisfactory, respectively: 0.86, 0.82, 0.84, 0.85 for the subscales, and 0.88 for the Global infertility-stress measure. Only the global measure of infertility-related stress was used in the analysis of the current study.

#### 2.3.3. Positive body image

Positive body image was assessed using the Body Appreciation Scale-2 (BAS-2; Tylka and Wood-Barcalow, 2015), a validated unidimensional scale measuring the individuals’ acceptance, positive opinions, and respect for their bodies. The BAS-2 is composed of 10 items based on a 5-point Likert scale, ranging from 1 (never) to 5 (always). An example item is, “I feel good about my body.” A total score was calculated by summing the individual responses, with higher scores designating greater positive body image. The Italian version of the instruments has shown adequate psychometric properties (Casale et al., 2021). Cronbach’s alpha in this study was 0.96.

### 2.4. Data analyses

As a first step, we calculated descriptive analyses and zero-order Pearson’s correlations among study measures to verify the relations between attachment orientations, infertility-related stress, and body image. Two-tailed *p*-values <0.05 were considered statistically significant.

Then, we tested the associations between infertility-related stress and positive body image with several sociodemographic variables (age, years of education, relationship duration, duration of current cohabitation, duration of infertility, and infertility diagnosis), to identify potential confounders. Significant confounders (*p* < 0.05) were entered in subsequent path analyses as covariates.

**Figure 1:**
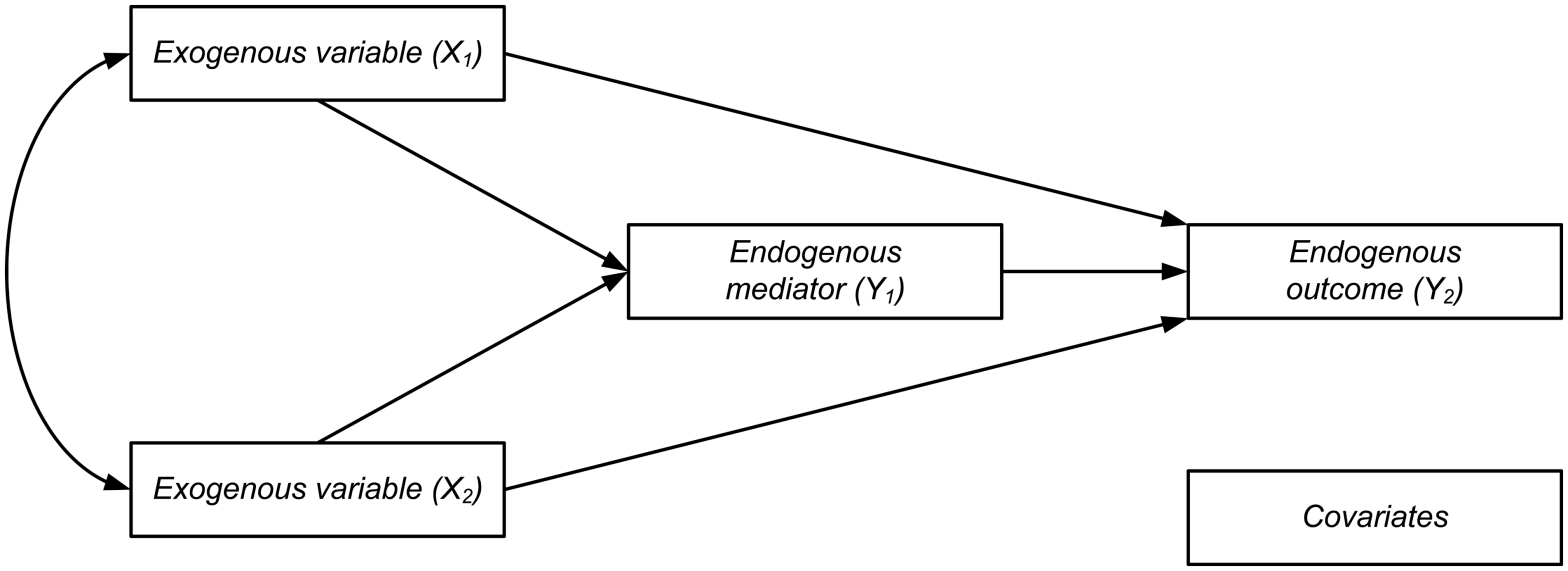
The conceptual diagram of the mediation model.

To verify the hypothesis that infertility-related stress may mediate the association of attachment with women’s positive body image, a path analysis was carried out. Attachment anxiety and attachment avoidance were included in the model (Model 1) as exogenous or predictor variables (*X*_1_, *X*_2_), and they were allowed to correlate, positive body image was considered as an endogenous outcome variable (*Y*_2_), and the global measure of infertility stress as the endogenous mediator variable (*Y*_1_). The conceptual diagram of the mediation model is represented in Figure 1.

Model fit was evaluated using the following criteria: root mean square error of approximation (RMSEA) ≤ 0.06, standardized root mean square residual (SRMR) ≤ 0.08, and comparative fit index (CFI) > 0.95 (Hu and Bentler, 1999).

To verify the mediation hypotheses, 95% percentile bootstrap confidence intervals (CI) for the indirect effects were calculated using 5,000 bootstrap samples. Confidence intervals of indirect effects not crossing zero were considered statistically significant and the corresponding mediation was established (Preacher and Hayes, 2008).

Lastly, we carried out a second path analysis to test an alternative, reversed model of mediation (Model 2). This model was similar to Model 1 but mediating and outcome variables were reversed: positive body image was treated as the mediator (*Y*_1_) and infertility-related stress as the outcome variable (*Y*_2_). Attachment anxiety and attachment avoidance, like in Model 1, were exogenous or predictor variables (*X*_1_, *X*_2_).

Model 1 and Model 2 were compared to select the best model using Akaike’s information criterion (AIC) and the Bayesian information criterion (BIC). The model with the lower value of AIC or BIC was considered preferable, indicating a better balance between model fit and model complexity (Lin et al., 2017).

All statistical analyses were performed using the IBM Statistical Package for the Social Sciences (SPSS 26) and Amos, version 24 (IBM Corp., Armonk, NY, United States).

## 3. Results

As a preliminary analysis, we computed descriptive statistics and Pearson’s correlations among the study measures. Attachment anxiety and attachment avoidance positively correlated with the global measure of infertility-related stress derived from the FPI-SF and negatively with the positive body image. As expected, infertility-related stress and positive body image were negatively related. According to Cohen’s (1988) conventions for statistical effects, all correlations showed a “medium” effect size (≥0.30) or were very close to it.

Descriptive statistics and correlation coefficients are presented in Table 1.

**Table 1:** Descriptive statistics and Pearson’s correlations among study measures and potential confounders.

Measure	*Theoretical score range*	*M*	*SD*	1	2	3	4
1. Attachment anxiety (ECR-R)	18–126	49.74	19.02	–	0.50**	0.32**	−0.34**
2. Attachment avoidance (ECR-R)	18–126	44.45	19.31		–	0.39**	−0.27**
3. Infertility-related stress (FPI-SF)	27–162	98.78	23.76			–	−0.29**
4. Positive body image (BAS-2)	10–50	35.29	9.43				–
Age		36.7	6.0			−0.17	0.09
Years of education		15.2	3.7			0.03	0.05
Relationship duration		9.9	5.8			−0.08	0.06
Duration of current cohabitation		6.9	4.5			−0.21*	0.04
Duration of infertility		4.2	4.0			−0.11	0.03

M: mean; SD: standard deviation; *p < 0.05; **p < 0.01.

Using Person’s correlation analysis, we tested the associations between infertility-related stress and positive body image with age, years of education, relationship duration, duration of cohabitation, and duration of infertility of the participants to identify potential confounders. Three participants were excluded from the analysis because of missing data.

All the correlations were not significant (*p* > 0.05) except for the duration of current cohabitation, which was negatively correlated with infertility-related stress (*r* = −0.21, *p* < 0.05). Correlations are reported in Table 1. Two ANOVAs were computed to verify differences in the scores of infertility-related stress and positive body image related to an infertility diagnosis. The results were not statistically significant [*F*(3, 109) = 1.61, *p > 0*.05; *F*(3, 109) = 0.26, *p > 0*.05, respectively].

Consequently, the duration of current cohabitation was entered in subsequent path analyses to take into account the potential confounding effect of this variable on infertility-related stress (the total number of participants included in the analyses was reduced to 110).

An *a priori* power analysis was conducted to estimate the minimum number of participants needed to attain a power of 0.80 at alpha 0.05 for a simple mediation analysis under the assumption of medium indirect effects. A Monte Carlo power analysis for simple mediation models, conducted with the app developed by Schoemann et al. (2017), established that a minimum number of participants of 105 (with 1,000 replications used in the simulation) was needed to identify a medium indirect effect ab (with standardized coefficients for a and b of 0.30) with a power of 0.80 and α = 0.05.

**Figure 2:**
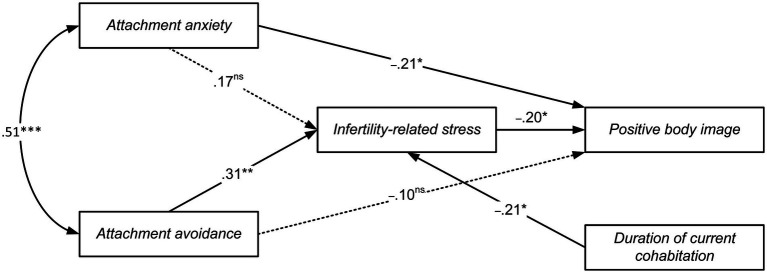
Path analysis of the relationship between attachment anxiety, attachment avoidance, and positive body image, as mediated by infertility-related stress. Path coefficients are standardized; Not significant paths are represented with dotted lines; ns = not significant; * *p* < 0.05, ** *p* < 0.01, *** *p < 0*.001.

The first mediating path analysis (Model 1) included attachment anxiety and attachment avoidance as predictors (*X*_1_, *X*_2_), infertility-related stress as the mediator (*Y*_1_), and positive body image as the outcome (*Y*_2_; Figure 2). The model showed an excellent fit to the data (χ^2^ [3, *N* = 110] = 0.532, ns; RMSEA = 0.000, SRMR = 0.014, CFI = 1) and accounted for 22 and 16% of the total variance of infertility-related stress and positive body image, respectively. AIC was 24.532 and BIC was 56.938.

Higher levels of attachment anxiety were directly associated with lower levels of positive body image (β = −0.21, *SE* = 0.005, *p* < 0.05) but not with infertility-related stress (β = 0.17, *SE* = 0.121, *ns*). On the contrary, attachment avoidance showed a positive association with infertility-related stress (β = 0.31, *SE* = 0.119, *p* < 0.01) but not with positive body image (β = −0.10, *SE* = 0.005, *ns*).

The analysis of indirect effects showed a significant mediating role of infertility-related stress in the link between attachment avoidance and positive body image (*b* = −0.003, 95% CI [−0.007, −0.001]): higher levels of attachment avoidance were associated with higher infertility-related stress, which in turn was significantly associated with lower body positivity image. The indirect link connecting attachment anxiety to positive body image *via* infertility-related stress was not significant (*b* = −0.002, 95% CI [−0.005, 0.000]).

A post-hoc power analysis, conducted with semPower (Moshagen and Erdfelder, 2016), indicated that Model 1, with an alpha of 0.05, an RMSEA of 0.05, and the current sample size, achieved a power of 0.90.

**Figure 3:**
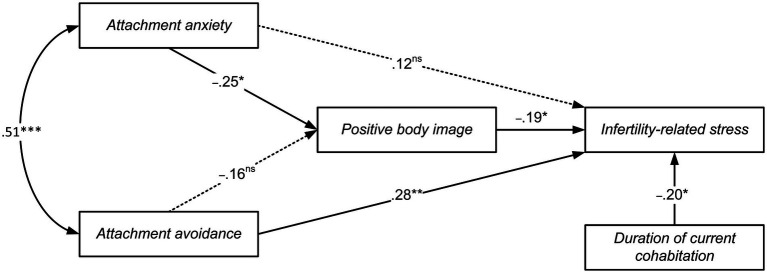
Path analysis of the relationship between attachment anxiety, attachment avoidance, and infertility-related stress, as mediated by positive body image. Path coefficients are standardized; Not significant paths are represented with dotted lines; ns = not significant; * *p* < 0.05, ** *p* < 0.01, *** *p < 0*.001.

Next, an alternative, mediating model (Model 2) was tested by carrying out a second path analysis. In this analysis, mediator and outcome variables were reversed: positive body image was treated as the mediator (*Y*_1_), and infertility-related stress as the outcome (*Y*_2_; Figure 3).

Again, the results showed a satisfactory model fit (χ^2^ [3, *N* = 110] = 0.577, ns; RMSEA = 0.000, SRMR = 0.017, CFI = 1). The explained variance was 25% for infertility-related stress and 13% for positive body image. AIC was 24.577 and BIC was 56.983.

Concerning the path coefficients, as portrayed in Figure 3, attachment anxiety showed a statistically significant negative direct effect on positive body image (β = −0.25, *SE* = 0.005, p < 0.05), attachment avoidance a positive effect on infertility-related stress (β = 0.28, *SE* = 0.118, *p* < 0.01), and positive body image a negative effect on infertility-related stress (β = −0.19, *SE* = 2.226, *p* < 0.05).

The indirect effect of attachment anxiety on infertility-related stress, mediated by positive body image, was significant (*b* = 0.056, 95% CI [0.010, 0.141]), whereas the indirect effect of attachment avoidance was not (*b* = 0.036, 95% CI [0.000, 0.120]).

To select the best data-supported model among Model 1 and Model 2, we used Akaike’s information criterion (AIC) and the Bayesian information criterion (BIC), as suggested by the literature (Lin et al., 2017). Model 1 had lower AIC and BIC values than Model 2. Therefore, Model 1 was considered the better choice in terms of balance between model fit and model complexity and was retained for interpretation.

## 4. Discussion

In this study, we examined the relationship between romantic attachment, infertility-related stress, and positive body image in women dealing with infertility. As expected, results indicated that both attachment anxiety and avoidance were significantly correlated with higher levels of infertility-related stress and with lower degrees of positive body image in women dealing with infertility. Higher levels of infertility-related stress were associated with lessened positive body image.

Mediational analyses showed a more complex picture, unfolding new aspects of the interplay among the constructs investigated in the study. When the mediating role of infertility-related stress was taken into account with the path analysis, only attachment anxiety showed a direct negative association with positive body image, whereas avoidance did not. In other words, results indicated that attachment anxiety of women facing infertility issues may increase their stress related to infertility and lower their positive body self-perception. These two associations, however, do not appear to be intertwined in a mediating relationship.

These findings are consistent with the existing literature. The significant association of attachment anxiety with infertility-related stress has already been documented in women undergoing fertility treatments (Donarelli et al., 2012). The negative link of attachment anxiety with body image is in line with those studies that found, in the general adult population, that only attachment anxiety seems to significantly predict negative body experience, satisfaction, and appreciation (Cash, 2004; van den Brink et al., 2016) or with those how reported that attachment anxiety is more important than avoidance in doing that (McKinley and Randa, 2005). This seems also compatible with Brennan and Shaver (1995) who found, using a different measure of romantic attachment, that body dissatisfaction was positively associated with the anxious-ambivalence rating but not with the avoidance one.

According to Cash (2011) an interpersonal insecure attachment system, such as the anxious one, may foster dysfunctional body image attitudes, including negative dispositional body image evaluations and lower degrees of body image investment. Body image evaluation indicates the satisfaction or dissatisfaction of individuals with their body and their beliefs about it. Body image investment refers to the cognitive, behavioral, and emotional importance ascribed to the body for self-evaluation. Both body image attitudes are thought to be central organizing constructs in the interplay of cognitive, emotional, and behavioral processes occurring in the context of interpersonal events. This aspect may be particularly relevant in the context of infertility treatments, when women’s bodies take center stage, regardless of diagnostic status (Halcomb, 2018). The literature on attachment has consistently reported associations between individual differences in attachment-system functioning and people’s perceptions of self (Mikulincer and Shaver, 2016). Individuals with an anxious romantic attachment style seem to have internalized a lacking sense of self-worth, which is likely to be contingent on the acceptance and approval of others and on the defensive mental processes that distort the perception of reality (Mikulincer and Shaver, 2016). These people are described as chronically unsatisfied with their partner’s love, approval, and support and are inclined to take some of the blame for the partner’s perceived unreliable care (Shaver and Mikulincer, 2012). They also commonly ruminate about their feelings of worthlessness in the relationship, and these thought processes can intensify the cognitive accessibility of negative self-representations (Mikulincer and Shaver, 2016). Moreover, the clumsy attempts of anxious-attached individuals to increase psychological proximity to their romantic partners tend to reinforce their negative self-image, because they may often present themselves in incompetent, childish, and excessively dependent ways in an inept attempt to elicit care and support from the attachment figure (Mikulincer and Shaver, 2016). When their interpersonal needs are not met, they are likely to feel even worse about themselves and their bodies in particular (Tantleff-Dunn and Lindner, 2011). Although attachment orientations are not gender-specific (Moura-Ramos et al., 2017), previous research in the field of infertility showed gender differences in attachment strategies, with women showing a greater tendency to activate anxious-oriented schemas (Greil et al., 2010; Moura-Ramos et al., 2017; Salcuni et al., 2021), communicating and expecting support from others (Peterson et al., 2007; Peterson, 2015). This aspect could be linked to cultural issues, assuming that behavioral patterns related to gender role expectations shape the strategies that women use when threatening events activate the attachment system (Mikulincer and Shaver, 2011).

Results concerning romantic avoidant attachment appear to show a different path: the hypothesized relationship between attachment avoidance and body image seems to be significantly mediated by infertility-related stress, whereas no direct association between avoidance and body image was found. Higher levels of attachment avoidance of the participants may increase their stress-related infertility, more than attachment anxiety does, and this heightened stress may reverberate negatively on the body self-appreciation of women with infertility issues.

This finding seems to be consistent with the previous results showing that women with higher attachment avoidance reported more sexual and relationship concerns, and more overall infertility-related stress (Donarelli et al., 2012). Attachment avoidance was also found to be associated with poorer emotional adjustment to infertility in women attending infertility clinics (Mahajan et al., 2008). Along the same line, a recent study confirmed the primary role of attachment avoidance on infertility-related stress of women with infertility issues, using an actor-partner interdependence analysis (Donarelli et al., 2016). In detail, these authors found that wives’ infertility distress was related to their own and their husbands’ attachment avoidance, but not attachment anxiety.

Attachment literature has repeatedly indicated that attachment security is associated with appraising stressful events in less threatening ways and with lower levels of distress (Mikulincer and Shaver, 2016). On the other hand, attachment anxiety and attachment avoidance are both associated with heightened distress. Individuals with high levels of attachment anxiety are inclined to appraise stressful events in more catastrophic ways and to perceive themselves as less able to cope effectively with them (Mikulincer and Shaver, 2016). Avoidant individuals, instead, are described as being similar to secure individuals when appraising their coping abilities, which are perceived as adequate, and similar to anxious individuals when dealing with threat appraisals (Mikulincer and Shaver, 2016). Typically, avoidant people are thought to rely on avoidance-focused or distancing coping strategies to face various kinds of stressful situations (Shapiro and Levendosky, 1999; Marshall et al., 2000; Holmberg et al., 2011). These distancing coping strategies can engage individuals in thoughts or behaviors that distract or disengage them from the stressor or that orientate away from the threat (Lazarus and Folkman, 1984) and they may be individually beneficial (Peterson et al., 2006). However, studies have shown that avoidant individuals may also perceive stressful events as highly threatening, and react with negative emotional reactions and with emotion-focused coping, in particular when these stressful events are demanding and prolonged (Birnbaum et al., 1997; Shapiro and Levendosky, 1999; Berant et al., 2001). Mikulincer and Shaver (2016) have suggested that “under chronic, demanding stressful conditions, avoidant deactivating strategies seem to collapse, causing avoidant people to have even higher levels of distress than anxious people.” (p: 206).

According to this interpretation, infertility may constitute a demanding stressful condition eliciting in avoidant women higher levels of infertility-related stress, which in turn may be negatively related to their positive body image. The negative association of infertility-related stress and body image appears to be consistent with previous studies showing that body image is negatively associated with infertility (Ozen et al., 2019) and perceived stress (Murray et al., 2011; Ziser et al., 2019). However, to the best of our knowledge, this is the first study investigating the relationship between positive body image and perceived stress specific to infertility. Infertility-related stress is a construct that differs from other measures of perceived stress, because it focuses specifically on infertility, comprising several relevant domains of such experience (Newton et al., 1999); therefore, it can be much more revealing about how women may experience infertility.

The present study has some limitations to be considered when interpreting the results. First, the study is cross-sectional and does not allow for causal inferences on the relations between variables. Therefore, other theoretically anchored models could be developed. For instance, in the current study, we also tested an alternative mediating model, with positive body image as the mediator and infertility-related stress as the outcome. This model showed a very good fit with the data collected (almost identical to the model under discussion), but it did not represent the better choice in terms of balance between model fit and model complexity. Hence, further research is needed to examine it. Another alternative model could explore whether heightened stress levels predict more attachment insecurity, which in turn could be linked to lower levels of positive body image. Or, it could also be conceivable that a low positive body image may heighten infertility-related stress and, at the same time, insecure attachment orientations. Future research should address these issues by collecting and analyzing longitudinal data and confronting different models of causal influences. Second, it is likely that the number of participants in this study was not large enough to statistically detect small to medium indirect pathways. Future studies should increase the sample size to overcome this limitation. Third, we used self-report measures: given the complexity of body experience in women facing infertility, future studies should integrate our findings with qualitative methods to improve an in-depth understanding of body image for infertile people. Specifically, the exploration of the point of view of those who live these experiences first hand could guide further research and clinical interventions in this field. In addition, although self-report measures are largely applied in epidemiologic research and are often considered more cost-effective (Jung et al., 2021), another limitation concerns the very self-reported nature of infertility-related characteristics collected (especially regarding the diagnosis). Moreover, our study employed an online data collection survey, which, as previously shown by scholars in this field, has some limitations regarding the self-selection of participants and their unknowability by the researcher (Janssens and Kraft, 2012; Andrade, 2020). Nevertheless, this method of data collection made it possible to reach a good number of participants and to develop a model of potential associations among the constructs investigated that we hope will be replicated and deepened by further studies. Besides, the current study included only female partners of heterosexual couples dealing with infertility. Future research should also examine male partners and investigate the cross-partner effects. Our study did not differentiate between women with primary or secondary infertility. Notwithstanding previous literature outlining that secondary infertility may represent a condition as stressful as primary infertility (Sormunen et al., 2018), future research should also explore possible differences within these groups in terms of positive body image. Finally, the current study did not take into account whether participants were receiving a MAR treatment, and what kind of treatment, at the time of the data collection. This issue should be considered a major limitation of the study and should be addressed in future replication studies.

Despite these limitations, the findings are consistent with expectations and contribute to knowledge in the area of attachment and positive body image in infertile women. Overall, the present study highlights the importance of romantic attachment orientations and infertility-related stress in influencing the body appreciation of women dealing with infertility. Our findings suggest two distinct paths in the relationship between romantic attachment and the body image of infertile women. The first path suggests that attachment anxiety may be negatively associated with body image in a direct way, and the second that infertility-related stress may mediate the negative association between attachment avoidance and body image.

Based on our results, future studies could focus on possible practical applications of these findings. It might be useful to develop short interventions designed to improve the psychological well-being of infertile women by reducing the negative consequences of attachment insecurities and infertility-related stress. Following and integrating the indications of Donarelli et al. (2012), short-term counseling interventions could focus on supporting infertile women to explore attachment dynamics with their partners, reinforcing their positive body image, and reducing infertility-related stress.

## Data availability statement

The raw data supporting the conclusions of this article will be made available by the authors, without undue reservation.

## Ethics statement

The studies involving human participants were reviewed and approved by Ethical Committee Psychological Research at the University of Padova. Written informed consent for participation was not required for this study in accordance with the national legislation and the institutional requirements.

## Author contributions

VC and CF: study design and conceptualization. VC: statistical analysis and data interpretation. VC, CF, CP, and CM: manuscript drafting and revision. All authors contributed to the article and approved the submitted version.

## Acknowledgments

The authors are thankful to the women who participated in this study.

## Conflict of interest

The authors declare that the research was conducted in the absence of any commercial or financial relationships that could be construed as a potential conflict of interest.

## Publisher’s note

All claims expressed in this article are solely those of the authors and do not necessarily represent those of their affiliated organizations, or those of the publisher, the editors and the reviewers. Any product that may be evaluated in this article, or claim that may be made by its manufacturer, is not guaranteed or endorsed by the publisher.
